# Towards the OR of the future: introducing an adaptive and technology-embracing OR wing layout

**DOI:** 10.1007/s11548-022-02760-7

**Published:** 2022-10-05

**Authors:** Carlos Amato, Chengyuan Yang, Lukas Bernhard, Pier Cristoforo Giulianotti, Paul Kondrat, Osman Ratib, Dirk Wilhelm

**Affiliations:** 1 CannonDesign, Los Angeles, CA USA; 2 Research Group MITI, Klinikum rechts der Isar, Technical University Munich, Munich, Germany; 3 Division of Minimally Invasive, General and Robotic Surgery, University of Illinois, Chicago, IL USA; 4 Department of Radiology and Medical Informatics, University Hospital of Geneva, Geneva, Switzerland; 5 Department of Surgery, Klinikum rechts der Isar, Technical University Munich, Munich, Germany

**Keywords:** OR of the future, OR design, OR planning, OR innovation

## Abstract

**Purpose:**

Overageing and climate change cause a need for making processes in the operating room wing (OR wing) more efficient. While many promising technologies are available today, traditional OR wings are not designed for seamlessly integrating these aids. To overcome this discrepancy, we present and motivate multiple ideas on how to transform current architectural design strategies.

**Methods:**

The presented concepts originate from expert discussions and studies of the available literature, but also from experiences made in the course of daily care delivery. Additionally, a comprehensive evaluation of current and historic OR theatre designs and the problems which are encountered herein has been conducted.

**Results:**

We present three innovative concepts regarding the restructuring of traditional OR wing layouts. To achieve better process optimization, hygiene, and energy efficiency, we propose to divide the OR wing into separate “patient”, “procedure” and “staff” zones. For better flexibility regarding perioperative needs and technology integration, we propose to use a hexagon shape combined with reconfigurable walls for designing operating rooms.

**Conclusion:**

The concepts presented herein provide a solid foundation for further considerations regarding perioperative process optimization and seamless integration of technology into modern OR wing facilities. We aim at expanding on these results to develop a comprehensive vision for the OR wing of the future.

## Purpose

**Fig. 1:**
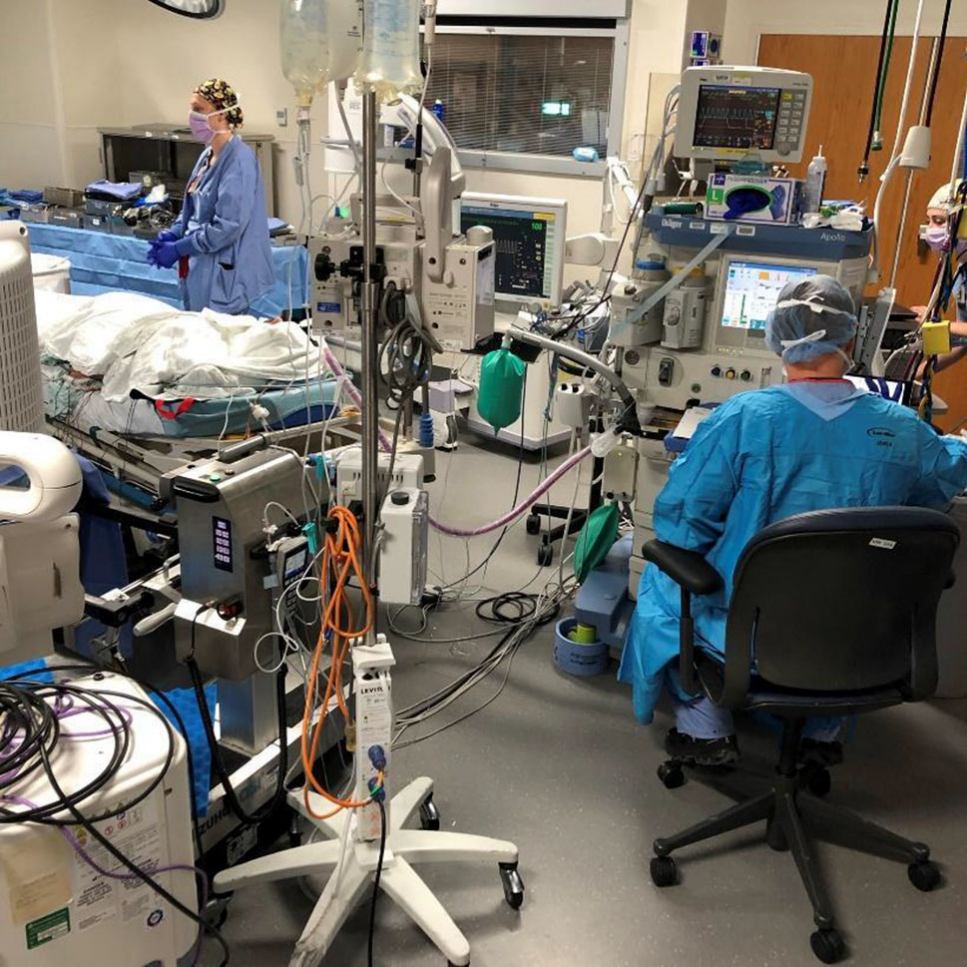
Today’s typical OR work conditions

The constant development of new interventional and surgical techniques, the demographic change leading to a mismatch between patient and staff numbers, but also the climate change and its request for reducing the CO_2_ emission, drive the need to rethink how operating room (OR) platforms are designed and operated. The main challenges we are facing here include the necessity to optimize staff utilization, the inflexibility of working spaces, the adherence to multifunctional rooms which are equipped with technologies mostly by demand and the low efficiency of usage of these costly and complex infrastructures (see Fig. 1). Therefore, in the future we are asked to do more with fewer people, while increasing the efficiency of OR usage at the same time. Reducing the amount of personnel required to run an OR in this regard will become the leading and most pivotal task. Irrespective of personnel constraints, the OR of the future—now more than ever—must offer a very specialized work environment, while being adaptive to changing procedure- and process-related requirements.

There are multiple key technologies that may prove pivotal in overcoming these challenges. Such means have been discussed and developed for several decades now [1–4], with the OR 2020 workshop being an important early milestone [5]. Various aspects of the OR of the future have been investigated in scientific literature, such as the integration and interoperability of medical devices and tools [5–15], visualization and intraoperative imaging [5, 7, 16], robotics [5–7], AI [17, 18], and simulation [19].

Clearly, all these aspects are central to the notion of the “OR of the future”. However, one further important and—in our view—severely neglected aspect is the optimization of clinical workspaces, both from an architectural as well as from a process-oriented standpoint. This proposition is strongly supported by work presented in [20–24]. While some efforts have been made to provide structured design methods for the operating room—most notably an OR layout optimization approach based on fuzzy constraint theory presented by Liu et al. [25]—there is a lack of truly disruptive and innovative concepts that enable fundamental improvement.

To that end, we envision breaking up the cellular design of current operating theatres in order to facilitate the sharing of tasks and infrastructure. In combination with a deliberate application of robotic technology (both remotely tele-manipulated and workflow assistive), there is great potential for reducing workspace demands, storage space and necessary transits of personnel involved in patient management and surgical interventions, in turn leading to more efficient workflows and a potential decrease of required building ground.

We are convinced we will have an increasing number of robotic systems in the future, which will reduce the need for surgeons to be present in the operating room. Surgeons can be located in special control rooms outside the sterile spaces, virtually anywhere in the hospital. However, current OR designs have not yet been adapted to this pending evolution. Furthermore, in non-robotic surgeries the number of assisting personnel will be reduced by minimally invasive approaches, by fewer extensive and salvage surgeries while moving to early stage and preventive interventions. Surgeries will be standardized to a much higher degree than today, and daylong complex surgeries will be replaced by multi-stage surgeries. All of these will increase the forecast reliability of surgical interventions and will lower the need for staff on demand, however, will increase the load of operations to be performed per unit and day. However, current OR designs focus more on creating the right conditions for a procedure than on optimizing the entire workflow, perioperative processes and a seamless transition from one operation to the next. Additionally, the pending introduction of mobile service robots, which will become a key element in this context, and their environmental requests yet need to be added to the architectural design.

Our work is an attempt to set future guidelines and strategies to deliver a new and enhanced surgical environment that is more efficient and cost effective, while improving the quality and accuracy of the procedures that are being performed. We achieve this mainly by rethinking the architectural design of the OR wing while taking into account procedural changes and technologies we already have or that will be available in a short while.

## Methods

**Fig. 2:**
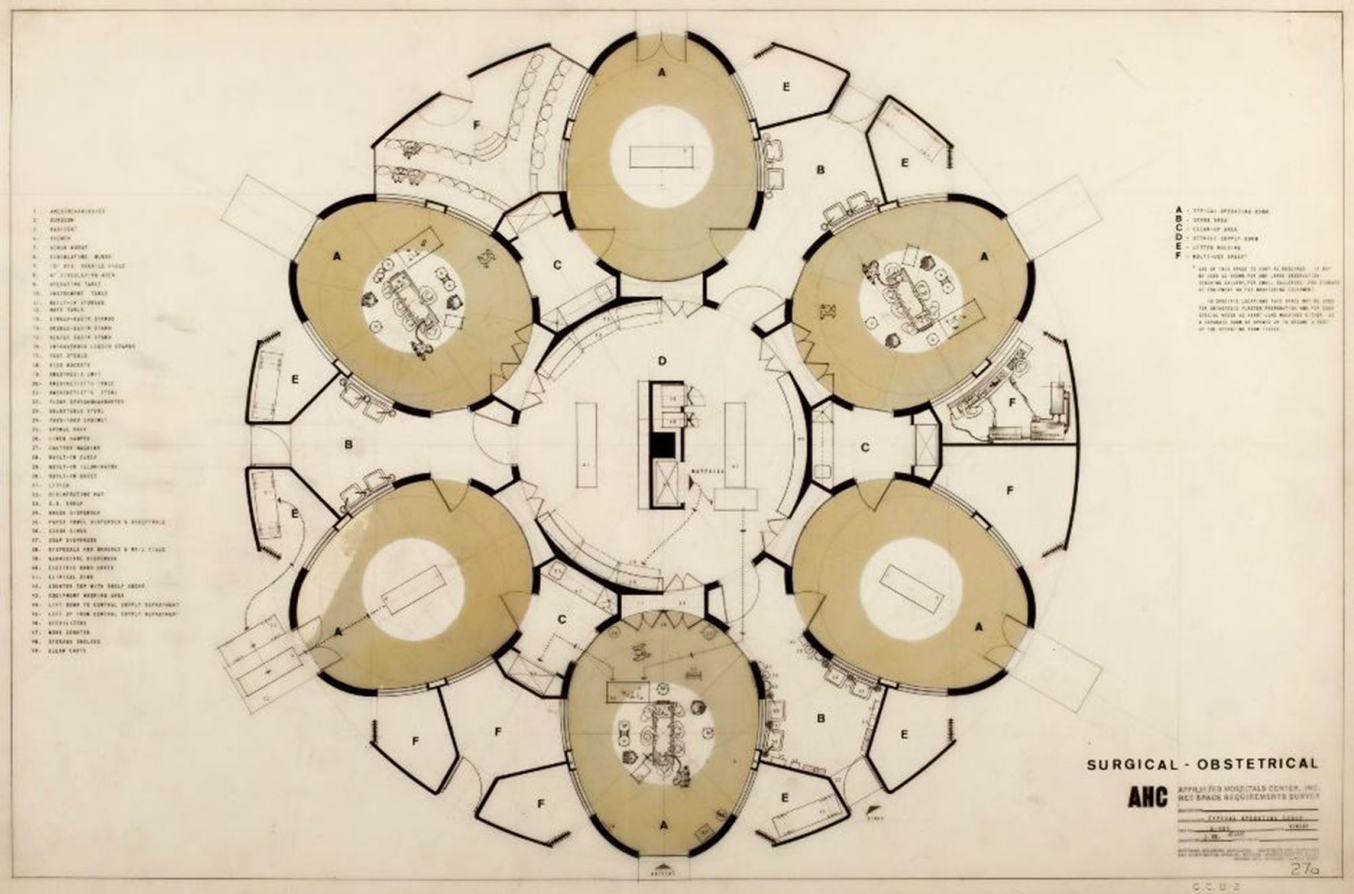
Surgery floor, Affiliated Hospital, Boston MA, 1964–1971

The results and thoughts which we present herein originate from expert discussions and studies of the available literature, but also from experiences made in the course of daily care delivery. Additionally, a comprehensive evaluation of current OR theatre designs and the problems which are encountered herein has been conducted. Further inspiration was drawn from historic design considerations, such as the concept of creating a concentric layout and placing critical support systems in the centre, which has several precedents from a time when none of the technology available today existed. These design explorations did not fully materialize but clued us to the benefits of considering such an approach (see Fig. 2).

The reflections made herein do also pick up experiences we made in course of the development of an autonomous mobile service robot for the OR (research project AURORA “Autonomous Self-Navigating Robotic OR Assistance”) and on computer simulations made in this context which we will present elsewhere (paper will be submitted to IJCARS/Conference proceedings). Also, aspects which were elaborated during the work on the patient hub concept [26] and have been debated in panel discussions on the OR of the future and on robots in healthcare are included. Still, the presented theses are speculative and visionary and thus cannot be fully based on a scientific background.

## Results

### Assumptions on pending changes in the OR

We have based our concepts and considerations on the following observations, assumptions and predictions regarding future developments of the surgical domain:

A. Shortage of personnel
B. Increasing number of performed interventions
C. Transition to more specialized surgeries and surgeons—potential separation of surgeries into parts that need to be handled by different surgeons
D. Increasing number of robotic and minimally invasive surgeries
E. Increasing number of supportive technologies (imaging, robots, navigation, etc.)
F. Change of individual workload by assistive technologies and AI—human dedicated tasks will move from actively involved to master control and action on demand
G. Higher demands on hygienic aspects, safety surgery and complication avoidance strategies
H. Reduction of procedure times and duration of interventions—more cases per OR suite—more transitions
I. Day-based surgery—patients will no longer be cared on a normal ward but will be directly be discharged from the OR
J. Constraints arising from the climate change—need for reduction of waste, for more economic work processes
K. Increasing number of hybrid surgeries which are involving different disciplines

These pending changes within the surgical OR result from the general evolution of surgery and from new technologies which have been introduced but have not yet been taken into consideration for the design of current OR units. Just as little does the architecture and the workflow of current OR areas support the further integration of such developments. Accordingly, we have rethought the design of the OR of the future and have identified three main innovations which correlate to these observations and which form a first approach in this regard.

### Innovation 1: Evolution towards an “on-stage” and “off-stage” surgical architecture

**Fig. 3:**
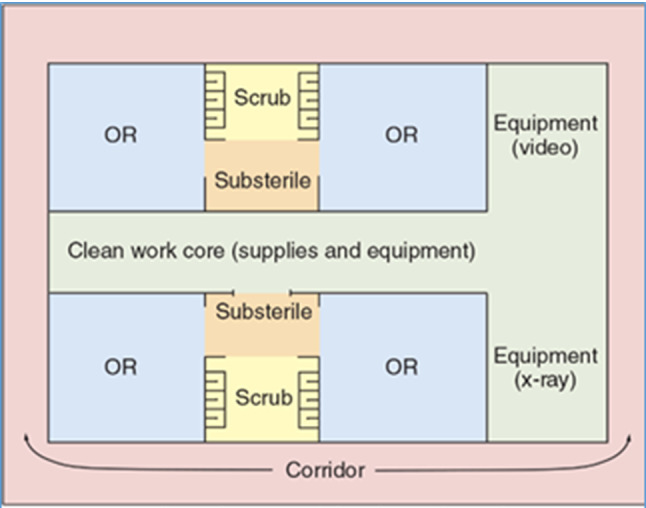
Traditional OR suite design (central core, peripheral corridor style)

**Fig. 4:**
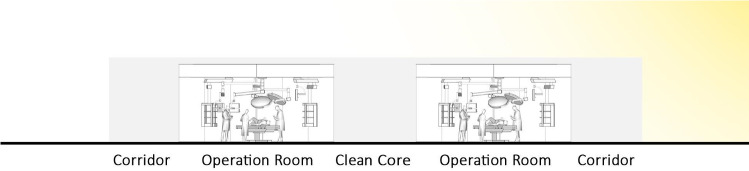
Traditional OR design cross section (central core, peripheral corridor style)

**Fig. 5:**
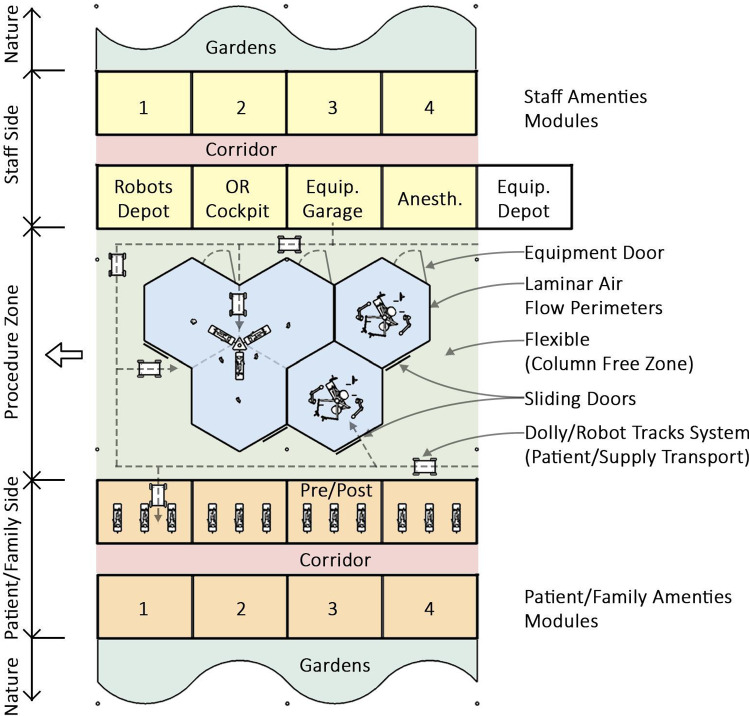
Re-invented OR design plan (two-sided on/off-stage concept)

**Fig. 6:**
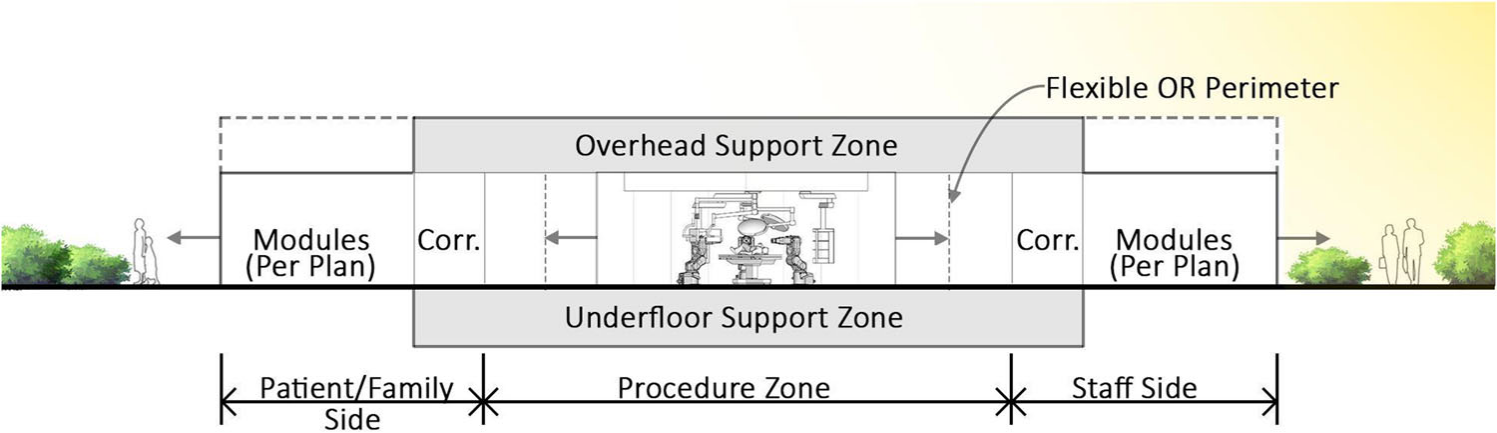
Re-invented OR design cross section (Two-sided on/off-stage concept)

Based on our investigations regarding traditional OR wing layouts (see Figs. 3 and 4), which spark many opportunities to rethink current processes, we advocate to change spatial qualities and allocating space for different purposes. In particular, we propose a two-sided, open-ended, radically adaptable layout with a central “procedure zone” (containing the operating theatres including prep-rooms), which is bookended with a “patient and anaesthesia zone” and a “staff and support zone” (see Figs. 5 and 6). Thus, we envision a clear separation of the traffic of personnel (and, in the future, robots and self-driving carts) on one side and patients and accompanying staff on the other side, meeting in the middle for conducting surgical procedures. Such an arrangement provides many opportunities for better adapting both sides to their individual needs, but also for improving hygienic aspects (perioperative sealing of OR against floor traffic and other patients) and safety issues (separation of patients from devices/technologies).

Via the patient and anaesthesia zone, patients will be brought into the OR and prepared for surgery, while this zone is also used for discharging patients after the completion of a surgery. Space for patient beds and waiting areas can be accommodated according to individual hospital needs and patient numbers. Access to the patient zone is only given prior and after the finalization of an intervention, sealing the OR theatre against contamination. The patient zone is adjoined to gardens and thus provides access to daylight and nature views, which has been linked to stress reduction and improved patient experiences and outcomes [27, 28].

On the other hand, the “staff and support zone” just opposite is dedicated to surgeons, operative assistants, mobile devices and supplies and is well separated from the patient side. This allows for a better optimization of these parts of the facility regarding the needs of the clinical staff and the integration of technology. For example, the integration of mobile service robots could be greatly simplified by not having to deal with patient/family traffic and by using dedicated and exclusive driving routes within the support zone.

We also propose to introduce remote surgery areas (RSA) for surgeons within the staff and support zone to provide a less confined and less cluttered environment while performing surgical interventions. RSA could also be helpful for training and assignment of surgeons to specific ORs, as experienced surgeons can easily switch between patients while training/assisting younger fellows. This is in contrast to the current situation, where surgeons have to change rooms and sometimes need to scrub-in, all of which is increasing the work load, negatively impacting the SSI rate and producing waste (gloves, coats).

We assume that improving the surgeon’s team comfort and quality of work environment would directly result in improved precision and efficiency of surgical procedures, in contrast to today’s environments which are aesthetically chaotic and often cluttered by equipment and instruments, which can interfere with the surgeon’s performance (see Fig. 1). The general consensus is that such improved environments can directly influence the outcome of the surgical procedures and even have an effect on patient recovery and survival rates. Furthermore, our two-sided layout offers the opportunity to place work and rest rooms adjacent to the main RSAs and ORs. These facilities—offering garden views and access to daylight—can be used by the medical staff in between procedures to take a break and refocus.

Innovation 1 addresses aspects B, C, D, E, G, H, I of the pending changes list.

### Innovation 2: Reshaping the OR wing layout to accommodate different types of interventions

Operating theatres rarely offer the perfect shape for a specific procedure and are, at least in defined phases of an operation, mostly perceived to be too small and too cramped. This aspect does not only change from one surgery to the other, but may also vary during the course of an intervention. While the operating room requires more space for navigating the patient and the technical equipment during preparation and transition phases, its floor plan can be significantly reduced during other phases of the procedure and, above all, at the end of the procedure. Furthermore, we need OR facilities to be flexible enough to integrate upcoming technologies, instead of having to adapt the technology to outdated buildings.

To address the need for short- and long-term flexibility of OR wing facilities, the two-sided surgery zone in between the patient and staff areas (see Innovation 1) is designed to allow the building structure and systems of each to be designed specifically and independently in response to their different functional needs. As uses and technology change over time, transformations can be accommodated in the surgery zone without impacting the other. To meet the structural requirements necessary for realizing this, we propose to support the central area on long-span trusses to maintain a column-free surgery floor plate while providing the equivalent of an interstitial floor for mechanical system routing and robotic transport systems. It also allows for underfloor support to accommodate robotic transport and other future systems. The areas surrounding the procedure zone and for spaces not requiring a column free arrangement are designed with a traditional universal column spacing.

The flexible and adaptive room design, which can also be changed during an operation if necessary, has numerous advantages. This results in a very economical use of space, as the available space can be optimally divided, which also appears favourable from an energetic point of view and the risk of contamination. The phase-dependent space requirement is also taken into account. For example, an operating room can be enlarged at the beginning of an operation, when the patient and the devices are brought in and these are connected to one another, and then reduced to the necessary minimum during the operation. In addition, this concept also includes the flexible use of the rooms according to the current situation and occupancy. A given room can be used, for example, as a preparation room, operating room or storage room, as required.

Reshaping working spaces and thereby optimizing workflows of personnel and technical instrumentations should allow us to accommodate different types of interventions by bringing in specific equipment on an “as needed” basis. Adaptive Reshaping space has proven to be superior for economic reasons and opens up an intervention space for upcoming technologies that would otherwise not be installable or would lead to a cramped environment.

One promising technology for the flexible delimitation of spaces are air curtains, which can be activated and deactivated to rapidly transform the layout of the OR wing according to current needs. This allows us to enlarge (or reduce) the size of the procedure and support spaces, reshape space to eliminate corners and turns, and combine functions for maximum efficiency. While mobile or even self-driving equipment (e.g., medical devices, service robots) is a promising technology in this context, we also consider to mount larger and bulkier devices to the ceiling and thereby free space on the floor level and reduce free-running cables, which otherwise would be obstacles for mobile equipment.

Innovation 2 addresses aspects B, C, D, E, J, K of the pending changes list.

### Innovation 3: Hexagon-shaped operating, anaesthesia and preparation rooms

Traditional operating rooms are commonly designed based on a rectangular floor plan, which we believe to be inferior in several regards. Firstly, a rectangular shape is not optimal for framing the sterile zone surrounding the patient, which often takes on more of an oval shape. As a consequence, not all of the available space is efficiently used, especially in the corners of operating rooms. Secondly, a rectangular floor plan is not optimal for clustering and interconnecting individual rooms, which is a prerequisite for a sharing and flexible reconfiguration of workspaces, as well as for achieving short paths for patients and personnel. We believe any person or human being who is in touch (or comes in contact) with a patient in the sterile environment (or field) increases risk of infection or errors. The more movement in and out, the higher the chances for infections and complications [29, 30].

Thus, instead of a rectangular layout, we propose to use the *hexagon* as the basic shape for designing operating rooms (see Fig. 5, centre). As motivated by mathematical considerations and supported by bionic observations (e.g. arrangement of honey combs inside a beehive), this shape requires shorter wall lengths for enclosing a given area, as compared to other geometries. While this reduces building costs and efforts, it is also inherently better suited for enclosing the sterile zone.

One of the main benefits becomes obvious when joining multiple hexagon-shaped units together: the amount of interconnections that can be made from one room to surrounding rooms is higher (six vs. four in the rectangular case), which benefits the implementation of shared workspaces and the co-location of patients. One example for this is the introduction of a shared anaesthesia unit, from which six surrounding operating rooms can be served by a single anaesthesia team. Similarly, preparation rooms and support spaces could be shared depending on specific needs and scenarios. For example, on the patient entrance side (see Innovation 1), patient preparation rooms can be designed to connect to one or multiple surgical rooms, depending on the duration of the surgery, case complexity and duration of the intervention. This allows for a more efficient workflow by limiting the waiting and deadtime between interventions while the patient is being prepared and set up for surgery. Preparing a patient while finishing ongoing interventions increases throughput and reduces times in which the available space is unused.

Pending changes A, B, F, J were considered when developing innovation 3.

## Discussion

Our “OR Wing of the Future” concepts presented herein provide a solid foundation for further considerations regarding perioperative process optimization and seamless integration of technology into modern OR wing facilities in order to provide the necessary prerequisites for full computerization, anaesthesia consolidation and automation, mobile and stationary robotics, patient transport automation and optimized supply and material distribution. Innovative architecture based on the three concepts introduced above has the potential to significantly impact the design and arrangement of OR suites in small and large healthcare facilities.

One future challenge, however, lies in developing tangible guidelines for designing and reconfiguring individual rooms, fitting in the necessary equipment, deciding where entry points should be, etc. Secondly, while the architectural design innovations which we did introduce herein are meant to enable the use of modern technologies, they also rely on them to become fully effective. While many of these technologies have already been realized and are about to be introduced to daily practice, they are faced with an outdated, rigid and technology reluctant environment. Thus, architecture and technology need to complement each other and, only when combined, can lead to exponential added value. Accordingly, the innovations outlined here must not be viewed solely from a design perspective nor as architectural brainstorming, but rather represent an urgently needed platform that enables the introduction and efficient implementation of innovative technologies.

However, it is important to stress that the “idea diagrams” included in this short communication paper are not intended to suggest a final design plan configuration and we do not claim the highlighted innovations to be complete. Instead, our ideas are supposed to stimulate additional concepts and multidisciplinary dialogue to improve surgical processes and outcomes.

## Conclusions

In recent decades, the technologies available for supporting surgical workflows have advanced and diversified tremendously, while the design of OR theatres remained mostly unchanged. The concept of current OR theatres is well suited for unplannable, exploratory surgeries, which are conducted by multi-specialized teams of different disciplines at the same time. However, this requirement is changing with the evolution of surgery and the operations performed today are increasingly conducted by highly specialized and technology-demanding experts, rather than by general surgeons, as in the past. We expect this evolution to continue and that, in the future, interventions will be performed in highly specialized OR suits by perfectly trained surgeons. With reference to hybrid ORs and robotic suites, this evolution has already begun.

It is therefore necessary to rethink current OR wing layouts and processes to achieve the necessary flexibility and to be able to seamlessly integrate the required technologies. In this short communication paper, we have motivated and formulated three core concepts that we believe are valuable building blocks for designing future-proof OR wing layouts. In future work, we aim at expanding on the presented concepts in order to develop an overarching vision and possible solutions for the OR wing of the future.
